# Differential dynamics of early stages of platelet adhesion and spreading on collagen IV- and fibrinogen-coated surfaces

**DOI:** 10.12688/f1000research.23598.2

**Published:** 2020-07-03

**Authors:** Melanie B. Horev, Yishaia Zabary, Revital Zarka, Simona Sorrentino, Ohad Medalia, Assaf Zaritsky, Benjamin Geiger

**Affiliations:** 1 Department of Immunology, Weizmann Institute of Science, Rehovot, Rehovot, 76100, Israel; 2 Department of Software and Information Systems Engineering, Ben-Gurion University of the Negev, Beer-Sheva, 84105, Israel; 3 Department of Biochemistry, University of Zurich, Zurich, CH-8057, Switzerland

**Keywords:** Platelet spreading, Microtubules, Integrin αIIbβ3, Type IV collagen, Fibrinogen, Interference Reflection Microscopy (IRM), Live cell imaging

## Abstract

**Background:** Upon wound formation, platelets adhere to the neighboring extracellular matrix and spread on it, a process which is critical for physiological wound healing. Multiple external factors, such as the molecular composition of the environment and its mechanical properties, play a key role in this process and direct its speed and outcome.

**Methods:** We combined live cell imaging, quantitative interference reflection microscopy and cryo-electron tomography to characterize, at a single platelet level, the differential spatiotemporal dynamics of the adhesion process to fibrinogen- and collagen IV-functionalized surfaces.

**Results:** Initially, platelets sense both substrates by transient rapid extensions of filopodia. On collagen IV, a short-term phase of filopodial extension is followed by lamellipodia-based spreading. This transition is preceded by the extension of a single or couple of microtubules into the platelet’s periphery and their apparent insertion into the core of the filopodia. On fibrinogen surfaces, the filopodia-to-lamellipodia transition was partial and microtubule extension was not observed leading to limited spreading, which could be restored by manganese or thrombin.

**Conclusions:** Based on these results, we propose that interaction with collagen IV stimulate platelets to extend microtubules to peripheral filopodia, which in turn, enhances filopodial-to-lamellipodial transition and overall lamellipodia-based spreading. Fibrinogen, on the other hand, fails to induce these early microtubule extensions, leading to full lamellipodia spreading in only a fraction of the seeded platelets. We further suggest that activation of integrin αIIbβ3 is essential for filopodial-to-lamellipodial transition, based on the capacity of integrin activators to enhance lamellipodia spreading on fibrinogen.

## Introduction

Mammalian platelets are small, anucleated megakaryocytes fragments that circulate in the bloodstream as single, smooth-surfaced discoid structures. Upon vascular injury, platelets are recruited to the wound, where they initiate the formation on the primary hemostatic plug, catalyze fibrin formation and facilitate wound healing. Their adhesion and subsequent spreading at the exposed extracellular matrix (ECM) play a key role in the healing process, which is modulated by a variety of external environmental factors such as additional plasma components, other ECM constituents, platelet-derived secreted factors and their specific membrane receptors ^1– 4^. In addition to the molecular composition of the environment, its physical properties, such as rigidity, topography and shear forces generated by the blood flow, also influence the platelets adhesion and spreading process.

These molecular interactions of the platelets with the microenvironment are governed by specific adhesion receptors, such as αIIbβ3 integrin, which bind to fibrinogen and participated in the formation of the fibrin clot, and α2β1 and GP-VI, that mediate platelet adhesion to collagen IV ^5– 9^. The molecular and physical cross-talk between platelets and their microenvironment was extensively investigated ^10– 14^, yet, given the complexity of the signaling pathways involved and their rapid activation, a comprehensive understanding of the platelet adhesion and activation process is still limited.

Previous studies have shown that platelets that are stimulated by a variety of agonists undergo radical morphological changes. The discoid shape, which is largely maintained by the circumferential marginal band of microtubules ^15– 17^, is affected by the agonists activation, and the cells acquire a spherical shape, extend filopodia and sheet-like lamellipodia ^18– 20^. The general process of platelet spreading on different surfaces was documented, yet how the platelets differentially respond to specific adhesive ligands is still unclear.

In this study, we compared the adhesion and spreading dynamics of isolated platelets on two distinct and physiologically-relevant ligands, namely collagen IV and fibrinogen, along a broad temporal scale, ranging from seconds to hours. We found that platelets in platelets-rich plasma (PRP-PL), and washed platelets (PLT) extend filopodia within a few seconds after reaching the close vicinity of fibrinogen or collagen IV surfaces, while still translocating along the surfaces. Stable adhesion to collagen IV or fibrinogen occurred roughly within a minute after filopodia extension, yet the mode of “immobilization” is clearly distinct; on fibrinogen the duration of “filopodial spreading” was shorter than on collagen IV (less than a minute, compared to ~2–3 minutes) and the onset of lamellipodia formation was apparent earlier (~1 minute, compared to over 2–3 minutes on collagen IV). It was nevertheless noted that only a fraction of the fibrinogen-adherent PRP-PLs proceeded to the lamellipodial spreading stage.

Unexpectedly, the delayed lamellipodial spreading on collagen IV (but not on fibrinogen) was commonly preceded by an extension of a single or a couple of microtubules from the circumferential marginal band to the platelet periphery, that penetrated into the core of peripheral filopodia. These results suggest that microtubule extension to the periphery of collagen IV-adherent platelets might trigger filopodia-to-lamellipodia transition, and promote platelet spreading.

## Methods

### Preparation of fibrinogen and type IV collagen surfaces

MatTek dishes were incubated with either 25 μg/ml human collagen IV (Sigma-Aldrich, Israel) in sterile PBS or with 50 μg/ml human fibrinogen (Sigma-Aldrich, Israel) in sterile PBS, overnight at 4°C. The human fibrinogen was rinsed twice in sterile PBS. Finally, blocking with BSA solution in sterile PBS (1mg/ml, 15 min incubation) was conducted, to reduce non- specific platelet attachment, followed by washing with PBS and with Tyrode’s buffer. The collagen IV was washed with DMEM- no additives (Sigma-Aldrich, Israel) for 3 × 30 min. To reduce acidity, it was then washed two times with Tyrode’s buffer. Both collagen IV and fibrinogen MaTek plates were subsided in Tyrode's-HEPES buffer pH 7.4 containing 5 mM dextrose/glucose.

### Preparation of platelets

Human platelets in platelet rich plasma (PRP-PL) were produced from freshly drawn blood of different healthy donors specifically for this study. As no Helsinki approval is required for such experiments, we have worked under the Weizmann Institute of Science Institutional Review Board (IRB) informed consent form, signed by each of the volunteers. The IRB application (375-1) was approved by the committee members.

The blood was collected into PT vacutainer tubes (containing sodium citrate). After 10 min of incubation at RT, the blood was centrifuged at 800 rpm for 10 min, and the supernatant, containing platelets and plasma was collected. Human washed platelets (PLT) were produced from freshly drawn blood of different healthy donors. The blood was collected into PT vacutainer tubes (containing sodium citrate). After 10 min of incubation at RT, the blood was centrifuged at 800 rpm for 10 min, and the supernatant, containing platelets and plasma was collected. Then an equal volume of Tyrode's solution pH 6.5 containing 5 mM dextrose, 0.2 µg/mL PGE _1_ and 1.0 U/mL apyrase was added and incubated for 5 min at RT, then the diluted PRP was centrifuged at 1500 rpm for 10 min to precipitate platelets. The supernatant was discarded and the pellet was re-suspended in Tyrode's solution, same volume as the discarded, at pH 6.5 containing 5 mM dextrose, 0.2 µg/mL PGE _1_ and 0.5 U/mL apyrase. The solution was incubated for 5 min at RT. Then it was centrifuged at 1500 rpm for 10 min. The supernatant was discarded and the pellet re-suspended in Tyrode's-HEPES buffer pH 7.4 containing 5 mM dextrose the same volume as the original PRP volume. The platelets were kept for 30–60 min at RT prior to use.

### Seeding of platelets on fibrinogen and collagen IV surfaces for IRM

Fresh PRP-PL or PLT were seeded (10–15×10 ^6^) on collagen IV and fibrinogen MatTek plates directly in the microscope environmental chamber at 37°C in humidified atmosphere of 5% CO _2_ and 95% air.

### Live-cell imaging and Interference Reflection Microscopy (IRM)

PRP-PL and PLT attachment and spreading process on the surfaces was monitored in real time using the DeltaVision Elite ^®^ system, running softWoRx 6.0. As the system is equipped with an environmental box, PRPs in Tyrode’s solution were seeded directly onto the fibrinogen and collagen IV surfaces in the microscope environmental chamber (temperature- and CO _2_-controlled). Interference reflection contrast (IRM) time-lapse imaging of several fields per specimen was carried out using 100x/1.3 oil objective, at 5 seconds between frames. IRM, phase-contrast microscopy, and differential interference contrast (DIC) microscopy still imaging were used with a 100X objective (NA 1.3 and 1.4), a 60X (NA 1.42) and a 20X (NA 0.85) objective.

### Spatiotemporal quantitative characterization of platelet spreading

Single platelets were manually selected in the field-of-view. Since we were interested in minimizing the effects of neighboring platelets, we selected platelets that spread the earliest in their vicinity. For each platelet, we manually annotated a bounding box that captured the full spread platelet and recorded time points for the characterized stages in platelet spreading (defined in Results): onset, or initial appearance (time = 0); onset of filopodia; and lamellipodia spreading.

To visualize and quantify the change in the local focal plane during platelet spreading, we developed custom software using Matlab and Python ( *Software availability* ^21^). We defined the *focal activity map* as the time-derivative of a platelet’s IRM intensity. This was calculated for each time point as ΔIRM _t_ = IRM _t_ – IRM _t+1_. Local instantaneous attachment was encoded with positive values (time point t+1 is darker – closer to the substrate), while local instantaneous detachment corresponded to negative values (time point t+1 is brighter – further from the substrate). The focal activity map provides intuitive visual information on the platelet’s spatiotemporal focal-dynamics.

The *integrated tapping activity* of a platelet in a given frame in the image sequence was defined as the accumulation of all local alterations in the focal plane, in both attachment and detachment events. This measure was calculated as the average value of the absolute ΔIRM image pixels: Σ _*i*_ | *IRM _t_*| _*i*_, where *i* spans the platelet’s bounding-box’s pixels, and | *IRM _t_*| _*i*_ is the corresponding pixel’s intensity (see Figure 2B). Importantly, this measure is relative; for example, more background pixels in a bounding box will dampen the magnitude regardless of the actual platelet activity.

To follow the spreading process, we quantified the pixels that were touching or very close to the surface. We pooled all IRM pixel intensities across a platelet time-lapse sequence and used the Otsu algorithm ^22^ to define a threshold that labels each pixel at every time point into ‘foreground’ or ‘background’. ‘Foreground’ pixels had low IRM intensities and were thus touching or very close to the surface, we termed these pixels as “interacting with the surface”. ‘Background’ pixels had higher IRM intensities and marked local regions that did not touch the substrate. We measured the number of pixels interacting with the surface over time during the spreading process (see Figure 2C). For each pixel, at each time frame, we accumulated the number of transitions from interaction to not-interacting with the surface from time 0 and until that time point (see Figure 2D).

To examine the spatiotemporal statistics of a platelet spreading, we identified a subgroup of active pixels. First, we manually marked a background region, an empty region close to the platelet, collected and pooled ΔIRM pixel intensities in the background throughout the platelet spreading process. Second, we used the accumulated background pixel intensity statistics to define thresholds of one standard deviation above or below the mean background pixel ΔIRM intensity. The subgroup of pixels that exceeded these ΔIRM thresholds were the more active pixels in terms of attaching/detaching activity. We calculated the fraction of active pixels that attached or detached for each time frame (see Figure 3A–C) and then overlaid the active pixels color-coded as attaching or detaching back to the image plane (see Figure 3D and E).

### Treatments

Mn ^2+^ (250 μM), molecule sn528 (αIIbβ3 integrin inhibitor; 10 μM) or thrombin (0.015 U/ml) were added directly to the dishes containing fibrinogen or collagen IV in Tyrode's-HEPES buffer pH 7.4 containing 5 mM dextrose simultaneously as the fresh PRP-PL or PLT, which were seeded (10–15×10 ^6^) in the microscope at 37°C in humidified atmosphere of 5% CO _2_ and 95% air.

### Chemical fixation of platelets in solution

The platelets were seeded for 10–20 min on fibrinogen or collagen IV. Subsequently, they were fixed by Karnovsky fixative (2% paraformaldehyde, 2.5 % glutaraldehyde). The solution was shaken well and incubated for 30 min. Then glycerol was added, spun down gently, and washed with PBS. The platelets were then suspended in PBS and glycerol (1:9), and the solution closed between coverslip and slide and imaged by phase contrast microscopy.

### Treatment with Nocodazole

Nocodazole (10, 15 or 20 μM) was added to the platelets in solution immediately before seeding the platelets in the dish containing collagen IV in Tyrode's-HEPES buffer, pH 7.4, containing 5 mM dextrose. Platelets were seeded (10–15×10 ^6^) in the microscope at 37°C in a humidified atmosphere of 5% CO _2_ and 95% air.

### Treatment with Von Willebrand factor (VWF)

Von Willebrand factor (2400 IU) Heamate P 1000 (CSL Behring, GmbH, Germany) was used in different concentrations (1:2, 1:5, 1:10), all yielding the same results. VWF was added directly to the dish containing collagen IV in Tyrode's-HEPES buffer, pH 7.4, containing 5 mM dextrose, simultaneously with the fresh PLT, which were seeded (10–15×10 ^6^) in the microscope at 37°C in a humidified atmosphere of 5% CO _2_ and 95% air.

### Cryo-electron tomography

Platelets were seeded on collagen IV functionalized gold/silicon grids (R 1/4, 200 mesh, Quantifoli, Jena Germany). The grids were then vitrified by plunge freezing in liquid ethane and then immediately stored in liquid nitrogen until imaging. Data acquisition was performed using a FEI Titan Krios equipped with a quantum energy filter and a K2 Summit direct electron detector (Gatan, Pleasanton, USA). Tomograms were acquired with a magnification of 42,000× corresponding to a pixel size of 0.34 nm. The cumulative electron dose was ~70 electrons per ångström ^2^. Tomograms were reconstructed with the TOM toolbox software package ^23^.

### Data analysis

Statistical calculations were performed using Excel (version 14.6.8, 2011) and a STATISTICA ^®^ (version 13.1) software package, using the non-parametric Wilcoxon rank-sum test.

Image analysis was performed using MATLAB (version 9.3), Amira/Avizo (version 3.1), motion correction software, and Fiji (ImageJ).

## Results

We chose to study two preparations of platelets, isolated from human donor blood: washed platelets (termed here PLT) and platelets in platelet-rich-plasma (termed PRP-PL). To create and test physiologically relevant environments, we plated platelets on tissue culture surfaces coated with either fibrinogen, an integral component of the blood clot; or collagen IV, a basement membrane molecule that platelets adhere to upon vessel injury. It is noted that this adhesive set-up is not entirely 'clean', as platelets secrete multiple biologically active molecules (e.g. fibrinogen). Furthermore plasma components in the PRP-PL may affect the experiments (for further discussion, see below). In this study, we have employed real time microscopy (primarily interference reflection optics (IRM) or differential interference contrast (DIC) for monitoring platelet-substrate interaction.

### Temporal steps in platelets’ interactions with collagen IV and fibrinogen surfaces in a time scale of seconds to hours

**Figure 1.**
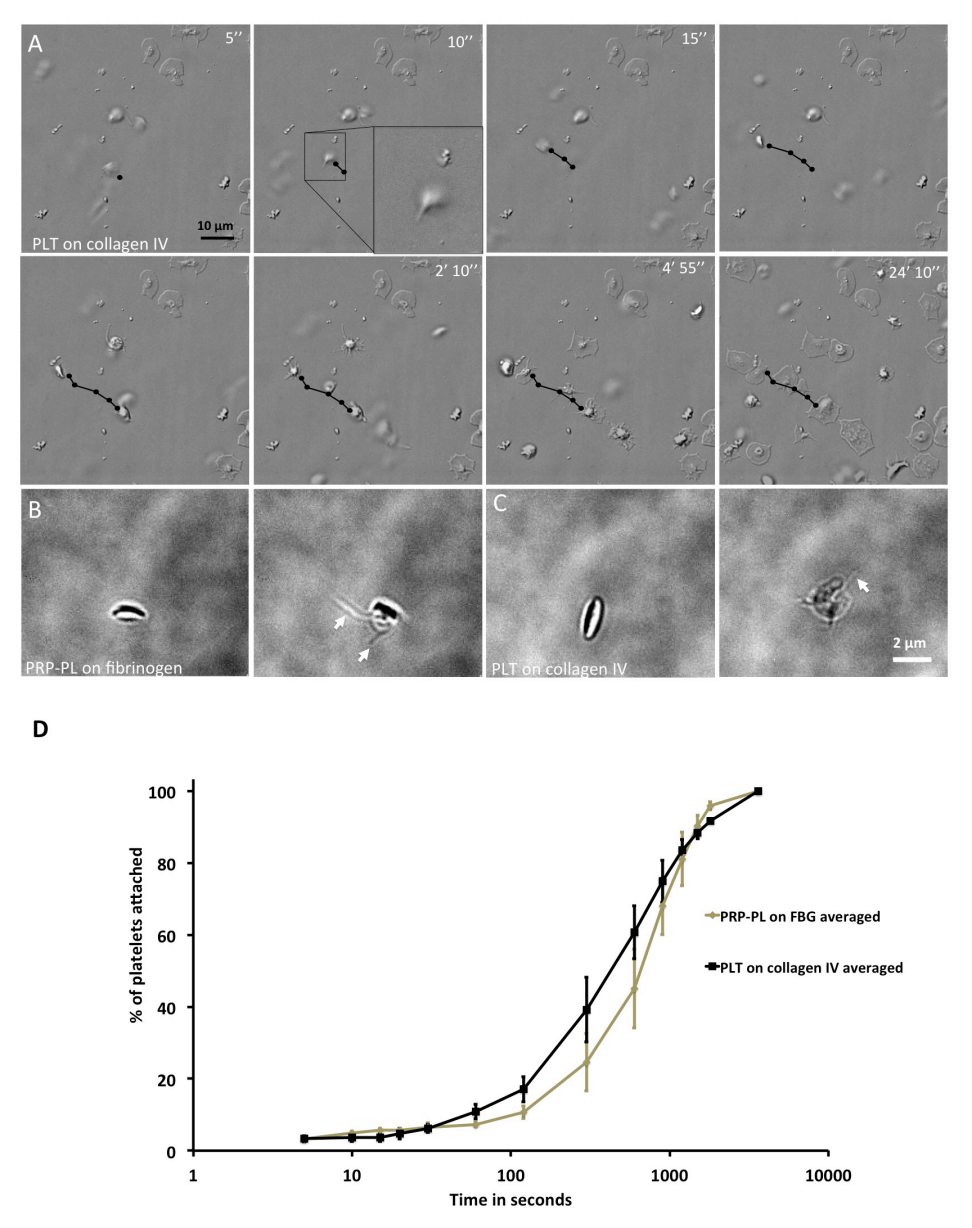
Observations of filopodia on platelets in solution on fibrinogen and collagen IV. ( **A**) DIC, time-lapse microscopy of platelets attaching to collagen IV at different time points. Black arrow denotes the platelet in question. Scale bar: 10 μm. ( **B**) Phase-contrast microscopy of PRP-PL on fibrinogen. Left: Discoid shape; Right: Platelet with filopodial extension. Scale bar: 5 μm. ( **C**) Phase-contrast microscopy of PLT on collagen IV. Left: Discoid shape; Right: Platelet with filopodial extension. Scale bar: 5 μm. ( **D**) Platelet attachment to collagen IV or fibrinogen in percentage (%) over time. Time in seconds, on a logarithmic scale. Platelets on collagen IV attach faster that on fibrinogen. Wilcoxon rank-sum test, p-value < 0.015. N platelets: FBG=121, collagen IV=274. Error bars show Standard Error.

*Pre-immobilization:* Early events of the interaction of platelets with collagen IV-coated surfaces were visualized using live DIC imaging. With the microscope focused on the adhesive surface, we observed platelets that were located at the vicinity of the substrate, whose translocation was substantially attenuated relative to those still floating freely in the medium. Such loose interactions with the surface were noted within 15–25 seconds after reaching the substrate focal level ( Figure 1A). We detected an active extension of “ventral filopodia” that was apparent as soon as 10 seconds after the platelet reached the surface ( Figure 1A, black arrow inset) and persisted until the platelet became immotile. Upon turning immotile, the platelet continued to spread by extending stable substrate-attached filopodia, followed by lamellipodia, until reaching full spreading after 20–30 minutes ( Figure 1A). We confirmed that some platelets extended filopodial extensions while being motile on collagen IV- or on fibrinogen-coated surfaces with chemical fixation and phase contrast microscopy imaging ( Figure 1B–C, arrows). These results confirmed the existence of a filopodial extension prior to platelets immobilization and attachment to the substrate.

**Figure 2.**
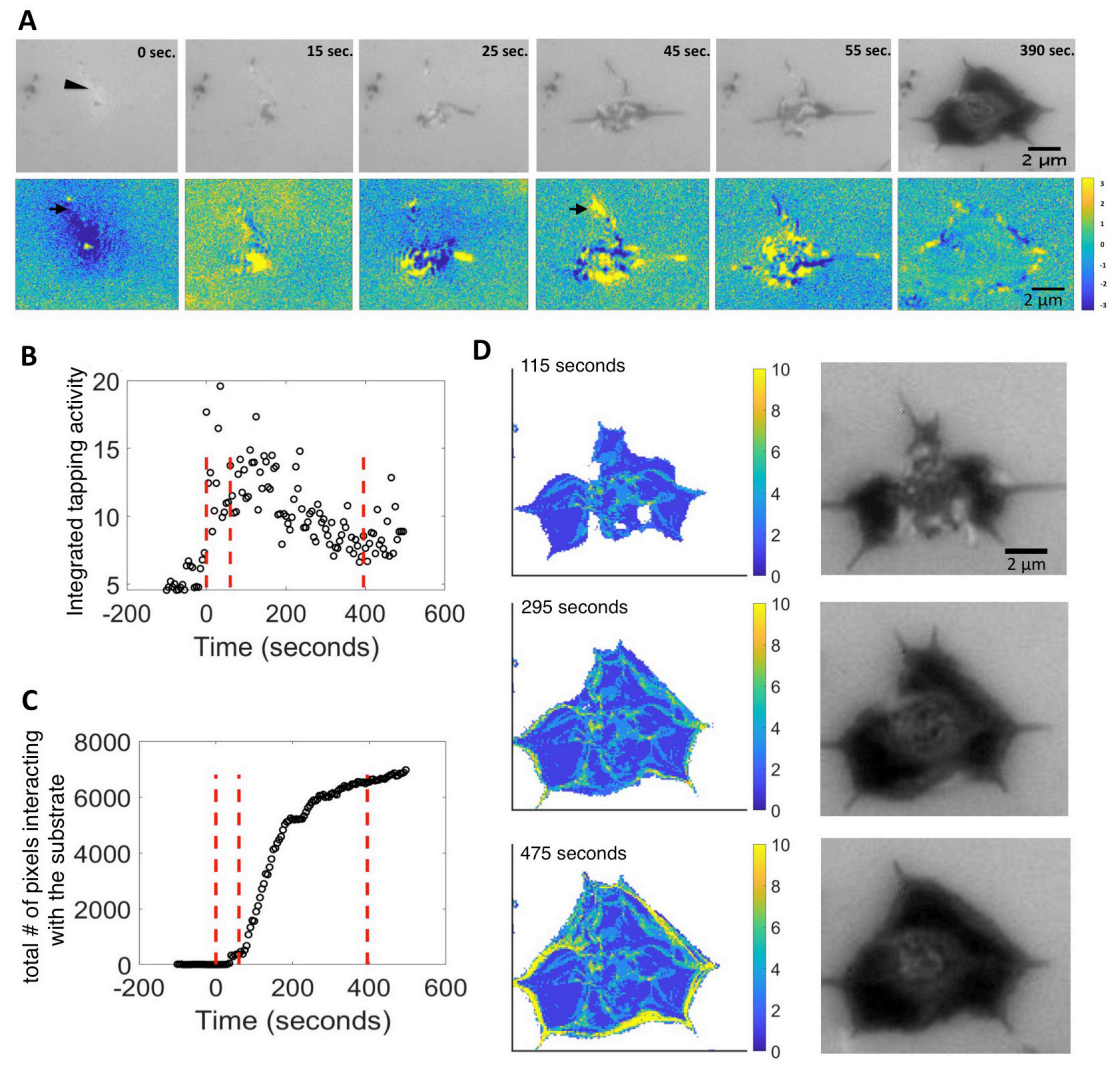
Quantification and image analysis of platelet spreading based on IRM live imaging for collagen IV. ( **A**) Platelet spreading viewed by IRM, and the corresponding focal activity map, ΔIRM _t_ = IRM _t_ – IRM _t+1_. Positive values (yellow) imply local attachment; negative values (blue) imply local detachment (bottom right). One filopodia initially attaching and detaching (black arrow). Scale bar 2 μm ( **B**) Integrated tapping activity of platelets: the mean absolute value |ΔIRM| at every time point. X-axis: Time in seconds. Y-axis: Platelet mean activity. Red dotted lines separate the phases: background, prior to platelet attachment, filopodial spreading phase, lamellipodial spreading phase, and the fully spread phase. ( **C**) Total number of pixels interacting with the surface over time. Time in seconds. ( **D**) Accumulated attachment and detachment events over time shown by activity map, yellow/blue means more/fewer events. Accumulated attachment and detachment events are adjacent to the color map. Images on the right correspond IRM images. Scale bar 2 um.

**Figure 3.**
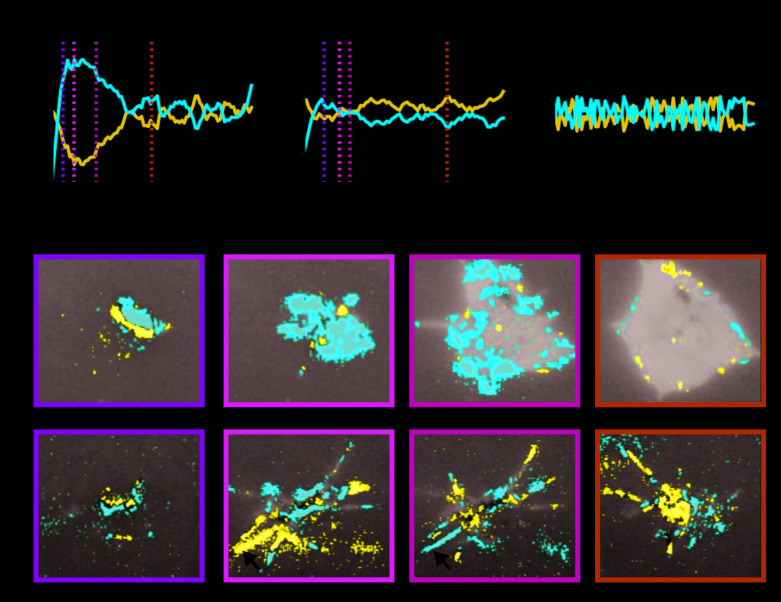
Differential platelet spreading: collagen IV versus fibrinogen. ( **A**– **C**) Temporal profile of the fraction of attachment (red) versus detachment (blue) events during platelet spreading: ( **A**) collagen-IV substrate, ( **B**) fibrinogen substrate, ( **C**) control – region with no platelet. ( **D** and **E**) Spatiotemporal dynamics of attachment (red) versus detachment (blue) events during platelet spreading. Time corresponds to the vertical dashed line in panels **A**– **B** correspondingly: ( **D**) Collagen-IV substrate. Platelet spreading is characterized by attachment, initially throughout the cell and later at the platelet periphery. ( **E**) Fibrinogen substrate. Platelet spreading is characterized by the attachment and detachment of entire filopodia structures (arrow heads).

*Post immobilization, filopodial spreading:* Platelets on collagen IV became immotile and attached to the surface at a faster pace than platelets on fibrinogen ( Figure 1D). These results suggest that upon the onset of platelet immobilization and spreading, platelets can extend filopodial protrusions on both collagen IV and fibrinogen surfaces, but that platelet spreading is more effective on collagen IV surfaces.

*Post immobilization, lamellipodial spreading:* The filopodial spreading, which was initiated still in the “mobile phase”, was followed by massive lamellipodial spreading (“filopodial-lamellipodial transition”), which was noted in the PRP-PL/fibrinogen system in ~55% of the platelets at about 1 minute after the first interaction with the surface. The onset of the filopodial-lamellipodial transition in the PLT/collagen IV system was delayed (~2–3 minutes) yet was apparent in a larger proportion of the platelet population ~75% (see results below).

### Live cell monitoring of the differential dynamic interactions of platelets with collagen IV and fibrinogen surfaces

To further characterize the spatiotemporal dynamics involved in platelet adhesion to collagen IV-coated surfaces, we used live cell IRM, where the light intensity is negatively correlated with the local platelet-surface proximity ( Figure 2A, top). To quantitatively characterize the spatiotemporal dynamics at the single platelet level, we manually cropped single images of platelets and analyzed them by measuring the pixel-wise difference in the IRM intensities between consecutive frames of the movie, imaged at temporal resolution of 5 seconds, ΔIRM _t_ = IRM _t_ – IRM _t+1_, which we termed the *focal activity map*. This analysis provided us with quantitative information and visualization regarding the local platelet dynamics: positive values (yellow) imply local attachment, and negative values (blue) imply local detachment, in relation to the previous time frame ( Figure 2A bottom). The brighter object observed at time = 0 was a characteristic feature of the IRM imaging for an unattached, but close to the surface object, and was consistently associated with a platelet appearing slightly above the IRM focal plane ( Figure 2A top, arrowhead). This out-of-focus feature provides the first visual cue for a near-future platelet attaching and spreading. Small attachment regions could be observed upon platelet appearance, at ΔIRM0 ( Figure 2A bottom, t = 0 sec., arrow). These local attachment regions corresponded to the future appearance of the first filopodia touching the surface ( Figure 2A, t = 45 seconds, arrowhead) and appeared in 18 of 20 platelets that we analyzed. This observation suggests that platelets sense the substrate from a μm-scale distance by extending thin protrusions toward the substrate that will later develop into stable filopodia. The appearance of full filopodia was characterized by local instability; some regions in the platelet were attaching to the substrate, while others were detaching concurrently, we termed this initial stage in platelet spreading, as the *filopodial spreading stage* ( Figure 2A, t = 45, 55 seconds). The filopodial stage was followed by a *lamellipodial spreading stage*, where local spatial instability was specifically observed at the platelet periphery, lamellipodia formed and platelet spreading preceded until reaching a fully spread morphology ( Figure 2A, t = 390 seconds).

To examine the platelet spreading process in a more quantitative manner, we developed a custom pipeline to analyze the local attachment/detachment dynamics in IRM time-lapse and applied it to platelets spreading on collagen IV surfaces. Briefly, we overlaid a 3×3 pixel grid of bins on the ΔIRM image, and collected temporal statistics on the attachment/detachment activity within these bins (see Methods). The overall attachment/detachment activity of a platelet, which we call the platelet’s *integrated tapping activity*, was defined as the mean absolute value |ΔIRM| at every time point ( Figure 2B). This measure constituted the integration of all local alterations in the focal plane, encompassing both attachment and detachment events. A platelet’s integrated tapping activity gradually increased during the filopodial stage, reaching a peak in activity, once this stage was complete. Platelet activity, then gradually decreased until it reached a plateau at the fully spread stage, which was above the initial integrated tapping activity before the platelet appeared. This was found to be consistent for most of the platelets measured ( *Extended data:* Figure S1 ^24^).

We next used a classical threshold-based segmentation method to separate the pixels of each time point into foreground and background pixels based on their IRM intensities ^22^ (see Methods). “Foreground” pixels had lower IRM intensities and were thus touching or very close to the surface, we termed these pixels as “interacting with the surface”. The number of pixels interacting with the surface over time showed a slow increase during the filopodial stage, and increased rapidly during the lamellipodial stage until reaching the fully spread morphology ( Figure 2C), this was consistent across several platelets ( *Extended data:* Figure S2 ^24^). By following the accumulated number of transitions from interaction to not-interacting with the surface at every pixel over time (see Methods), we observed that transitions occurred at the cell periphery as marked by no change in transitions accumulation in the platelet’s interior over time ( Figure 2D). The “vein”-like trails from the platelet center to its periphery suggest that the same filopodium is responsible for the repetitive interaction with the surface prior to the more stable lamellipodia ( Figure 2D; *Extended data:* Movie S1 ^24^). Altogether, these data suggested that on collagen IV platelets extend filopodia that interact with the substrate by local attachment-detachment tapping events. This filopodia sensing coincides with a steady increase in lamellipodial area, which eventually leads to overall platelet spreading on collagen IV. We observed similar results for platelets spreading on fibrinogen ( *Extended data:* Figure S3 ^24^).

On one hand, filopodia appeared during platelet immobilization on both collagen IV and fibrinogen surfaces. On the other hand, platelet spreading is less effective on fibrinogen surfaces that were characterized by impaired transition to lamellipodial spreading. To pinpoint how local spatiotemporal dynamics lead to global differential platelet spreading we identified the subgroup of pixels that undergo significant attachment/detachment activity (see Methods). By plotting the fraction of the active pixels that undergo significant attachment (red) versus significant detachment (blue) we observed that platelet spreading on collagen IV was initially characterized by rapid and dominating attachment ( Figure 3A). This is in contrast to the unstructured pattern that represents those platelets on fibrinogen that did not reach the lamellipodia-spreading phase ( Figure 3B) or control regions with no platelets ( Figure 3C). Overlaying these highly active regions on top of the IRM image revealed a striking differential pattern between platelet dynamics on collagen IV versus fibrinogen. While, platelets on collagen were characterized by attachment activity, initially throughout the cell body and later at the platelet periphery ( Figure 3D; *Extended data:* Movie S2 ^24^), platelets on fibrinogen were characterized by the attachment and detachment of entire filopodia structures ( Figure 3E; *Extended data:* Movie S3 ^24^). These results suggest that filopodia-to-lamellipodia transition is key for the effective spreading of platelets on collagen IV and less-efficient spreading on fibrinogen.

### Role of microtubules in platelets adhesion to immobilized collagen IV

**Figure 4.**
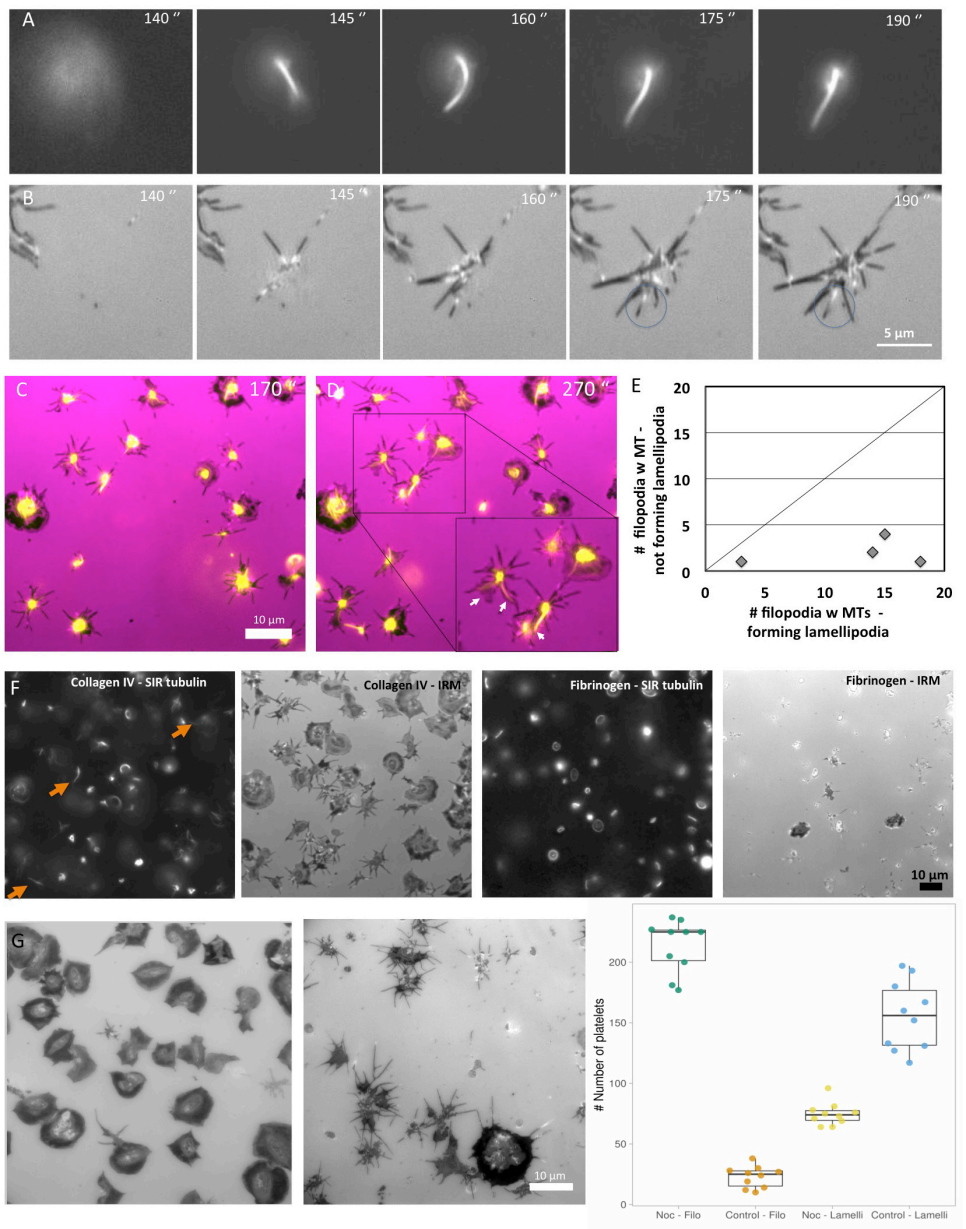
Platelet spreading and visualization of microtubule extensions. ( **A**) Platelets on collagen IV spreading every 5 seconds, as viewed by fluorescence microscopy (FM). Platelets tagged with tubulin are pictured in the upper row ( **B**) In the row below, platelets on collagen IV are imaged by IRM scale bar 5 μm. ( **C**) Platelet spreading on collagen IV at time point 170 sec. IRM (pink) overlay with SIR-tubulin (yellow). ( **D**) Same as ( **C**), at time point 270 sec. Lamellipodia formed where the SIR-tubulin extension is seen in filopodia (white arrows). ( **E**) Scatter plot showing each experiment, how many platelet filopodia with microtubules extensions exhibit lamellipodia formation. X-axis number of MT containing filopodia forming lamellipodia, y-axis number of MT containing filopodia not forming lamellipodia. N experiments=4 and n platelets=58. ( **F**) Washed platelets (PLT), on collagen IV and fibrinogen surfaces from left to right seen by SIR-tubulin and corresponding IRM, Sir-tubulin and corresponding IRM. Orange arrows indicate the dominant microtubule extension. Scale bar 10 μm. ( **G**) IRM images from time-lapse movies of platelets treated with/without nocodazole. Scale bar: 10 μm. Time point: 60 min. Far right: boxplot showing platelet specific shape – filopodia or lamellipodia spread, with and without nocodazole treatment. We saw that there was a difference between platelets treated with nocodazole compared to the control in terms of the number of filopodia spread platelets and lamellipodia spread platelets. Wilcoxon sum rank test was performed * p-value < 0.01, N experiments=10, n platelets = 4669.

A key step in platelet-substrate interaction is the transition from filopodial-to-lamellipodial spreading, which displayed different dynamics on the two surfaces (delayed by 1–2 minutes on collagen IV). In an attempt to understand the mechanism underlying the timing of this transition, we explored the cytoskeletal state of the platelets, particularly their microtubule (MT) dynamics by tagging live platelets with SIR-tubulin, which allows real-time monitoring of MT. These experiments clearly indicated that the vast majority (~94%, n=199) of platelets adhering to collagen IV commonly extend MTs towards the platelet periphery, where it interacts with and penetrates to a single, existing filopodium ( Figure 4A and B, time point 175 seconds). The extension was not confined to a primary filopodia, suggesting that it is not involved in filopodia formation per se. Further analysis of the platelet spreading showed that the early-protruding lamellipodium usually extended from the location of the main MT-targeted filopodium ( Figure 4C and E).

In platelets spreading on fibrinogen we did not observe microtubule extension into filopodia, suggesting that this phenomenon is collagen IV-specific. This observation, combined with the common failure of platelets adhering to fibrinogen, to undergo complete filopodial-to-lamellipodial switch, suggests that local microtubule extension to the platelet’s periphery plays a key role in the transition to lamellipodial spreading ( Figure 4F). This notion is further supported by experiments showing that treatment of PLT/collagen IV with nocodazole impaired lamellipodia formation ( Figure 4G), in agreement with 25.

**Figure 5.**
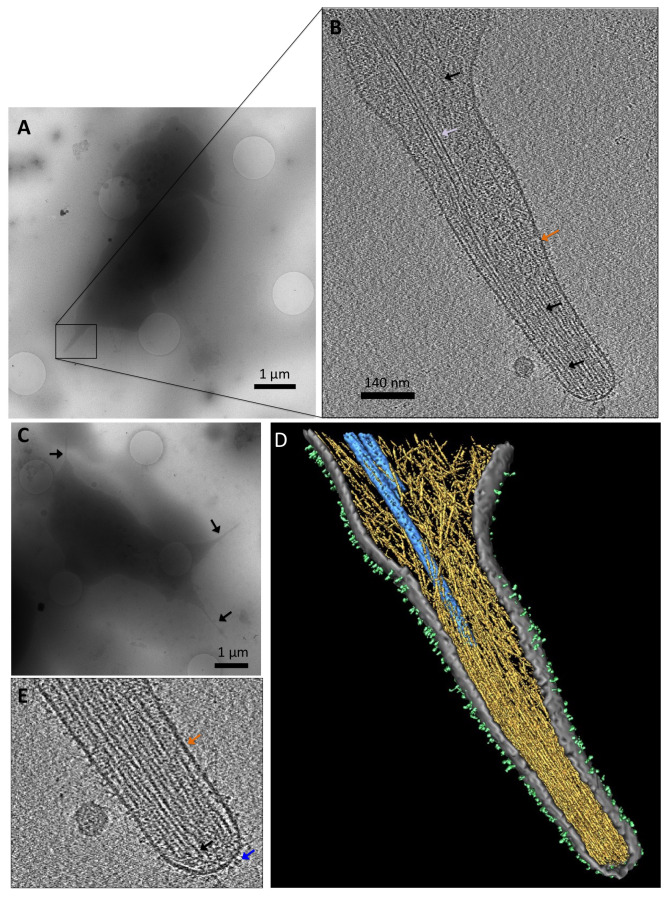
Cryo-electron tomography of a platelet filopodia on collagen IV. ( **A**) Low magnification image of a whole platelet with filopodial protrusions. Scale bar: 1 μm. ( **B**) The same platelet as in ( **A**), with a high magnification image of the filopodia in ( **A**), black box. Slice from the 3D reconstructed volume. Visible is the membrane (orange arrow), microtubules (white arrow), and actin (black arrows). Scale bar: 140 nm. ( **C**) Low magnification image of a representative platelet with filopodial extensions (black arrows). Scale bar: 1 μm ( **D**) Rendered volume of the whole tomogram. Actin in yellow, microtubules in blue, membrane in grey, and surface receptors, many are integrins, in light green. ( **E**) Zoom-in view of ( **B**), a slice from the 3D reconstructed volume. We are able to see the membrane (orange arrow), actin (black arrow), and surface receptors resembling integrins (blue arrow).

The MT-targeted filopodia from a random platelet revealed that microtubules entered the filopodia, but did not reach the tip of the filopodia as seen by cryo-electron tomography ( Figure 5A and B). We observed an abundance of actin filaments (black arrows) in the filopodia, but only 1–2 microtubules that entered the filopodial core. Overall, we located MTs in ~58% of the filopodia we imaged ( Figure 5C). From the 3D view we could appreciate the organized actin bundles at the tip of the filopodia, whilst more dispersed and organized in a network closer to the shaft ( Figure 5D). We also observe a plentitude of trans-membrane receptors ^26– 28^, many of who are integrins ( Figure 5E).

### Differential receptor-mediated surface recognition of PRP-PL and PLT, and their effects on the attachment and spreading on collagen IV and fibrinogen

**Figure 6.**
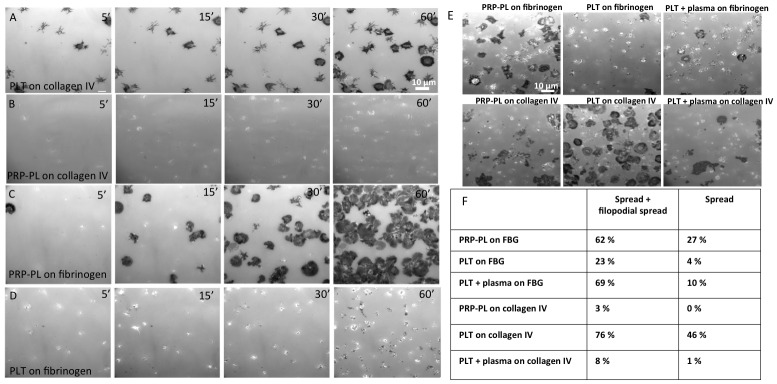
Platelet dynamics on fibrinogen and collagen IV surfaces. ( **A**– **E**) IRM imaging of platelet spreading for 1 hour. Scale bar: 10 μm. ( **A**) PLT spreading on collagen IV surfaces. ( **B**) PRP-PL spreading on collagen IV. ( **C**) PRP-PL spreading on fibrinogen. ( **D**) PLT spreading on fibrinogen. ( **E**) PRP-PL and PLT spreading on fibrinogen and collagen IV, with and without the addition of plasma to the PLT, 1 hour post-platelet seeding. ( **F**) Percentage of platelets in each morphological state, 1 hour post-platelet seeding.

When we first examined the interactions of both PLT and PRP-PL, with each of the two surfaces, striking differences in adhesion dynamics was found ( Figure 6A and B). In the experiments presented in the previous sections of this article we have primarily compared the adhesion and spreading dynamics of PLT on collagen IV to those of PRP-PL to fibrinogen. The primary reason for this pairing of platelet type (PLT and PRP-PL) and matrix type (collagen IV and fibrinogen), respectively, was that switching these platelets-matrix pairs resulted in poor-to-no adhesion and spreading. Specifically, PLT on collagen IV were characterized by little aggregation, adhesion followed by filopodial, and then lamellipodial spreading as described above (see also Figure 6A). PRP-PL, on the other hand, failed to attach and spread on collagen ( Figure 6B).

This behavior was essentially mirror-imaged by PRP-PL, which attached to fibrinogen surfaces and proceeded by extending filopodia. Some of these platelets further developed lamellipodia and progressed to the fully spread state, while others remained in the filopodia state ( Figure 6C). The attachment of PLT to fibrinogen was rather poor; only few platelets adhered to the matrix, and even fewer spread on it ( Figure 6D).

The failure of PRP-PL to adhere to collagen IV was attributed to von Willebrand factor (VWF), which was present in the plasma, and effectively block collagen IV binding. The blocking is caused because the VWF-platelet binding requires the A1 domain on VWF to be exposed in order to bind. The binding necessitates immobilization of VWF followed by shear force. Since our experimental condition are static VWF attaches to collagen IV, but does not bind the platelets ^29, 30^. It was further shown that the addition of VWF to PLT preparations blocked the attachment and spreading ( Figure 6E and F).

The limited ability of PLT to adhere and spread on fibrinogen is not entirely clear and could be attributed to the removal of VWF when the plasma is removed. VWF binding to integrin αIIbβ3 could play a role in this process ^31^.

**Figure 7.**
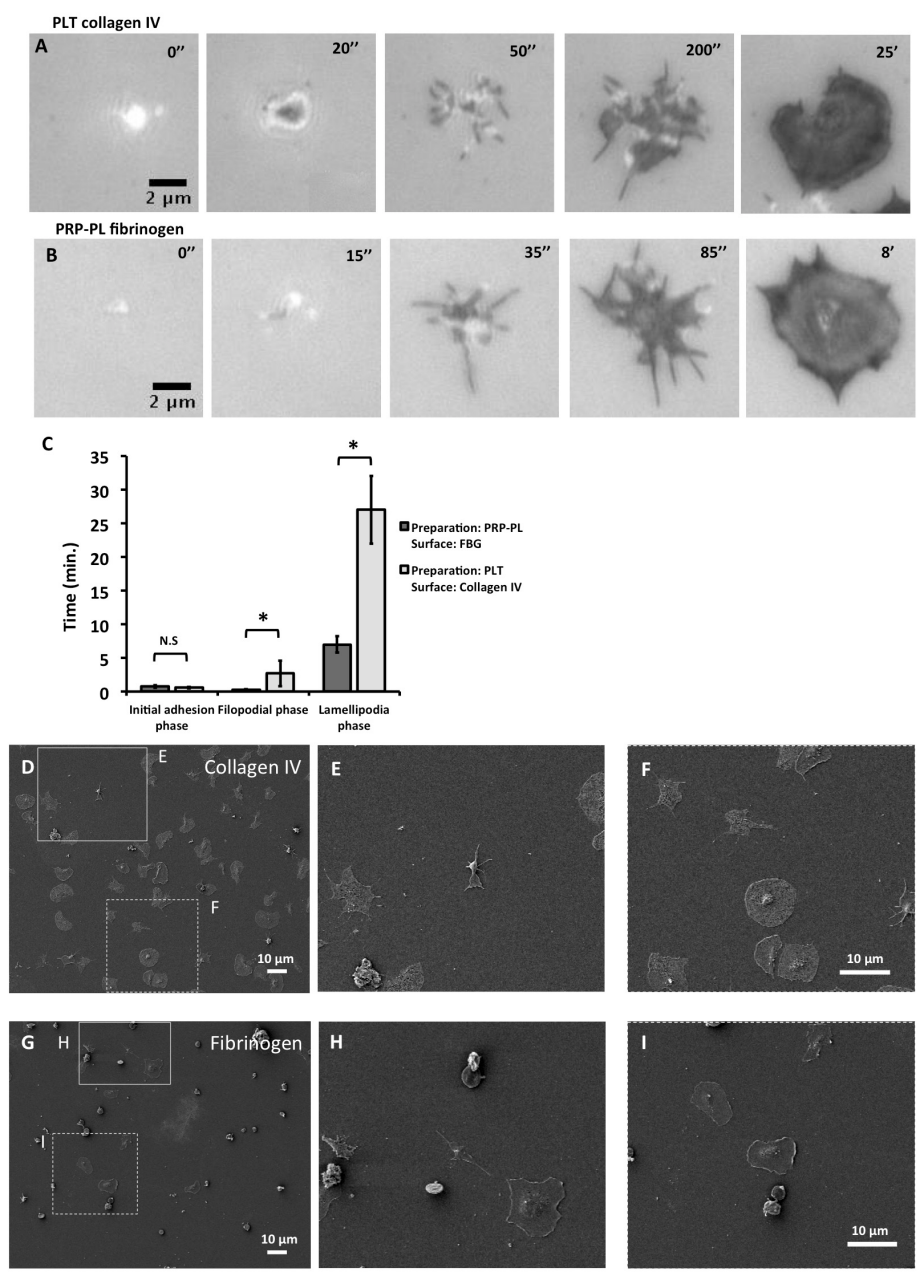
Use of IRM and SEM to image platelet dynamics on collagen IV and fibrinogen. ( **A**) IRM (100X) of a platelet (PLT) spreading on collagen IV; (left to right) filopodia tapping and fluctuations (0’’, 50’’), filopodia spreading (50’’, 200’’), lamellipodial protrusion (200’’), to the final, spread shape (25’). Scale bar: 10 μm. ( **B**) IRM (100X) of a platelet (PRP-PL) spreading on fibrinogen; (left to right) filopodia tapping and fluctuations (0’’, 35’’), filopodia spreading (35’’, 85’’), lamellipodial protrusion (85’’) to the final, spread shape (8’). Scale bar: 10 μm. ( **C**) Dynamics of platelet spreading. Graph showing the length of each of the phases described in ( **A**) and ( **B**). Wilcoxon rank sum test was used. *p-value < 0.01, N platelets collagen IV=40, N platelets fibrinogen =40. Error bars show Standard Error. ( **D**) SEM image (1.00 K X) of PLT on collagen IV. Scale bar: 10 μm. ( **E**) SEM image (1.00 K X) of PLT on collagen IV, showing a filopodia spread platelet. ( **F**) SEM image (1.00 X K) of PLT on collagen IV, showing a fully spread platelet ( **G**) SEM image (1.00 K X) of PRP-PL on fibrinogen. Scale bar: 10 μm. ( **H**) SEM image (1.00 K X) of PRP-PL on fibrinogen, showing a filopodia spread platelet. ( **I**) SEM image (1.00 X K) of PRP-PL on fibrinogen, showing a fully spread platelet. Scale bar: 10 μm.

The overall spreading rate of PLT on collagen IV, from initiation to maximal extension, was ~ 2.5-fold slower than that of PRP-PL on fibrinogen (10 minutes, compared to 25 minutes, Figure 7A and B). Platelets on collagen IV exhibited longer filopodia and lamellipodia spreading phases, than platelets on fibrinogen ( Figure 7C). The filopodia and lamellipodia spreading phases on both surfaces were demonstrated by scanning electron microscopy ( Figure 7D–I).

**Figure 8.**
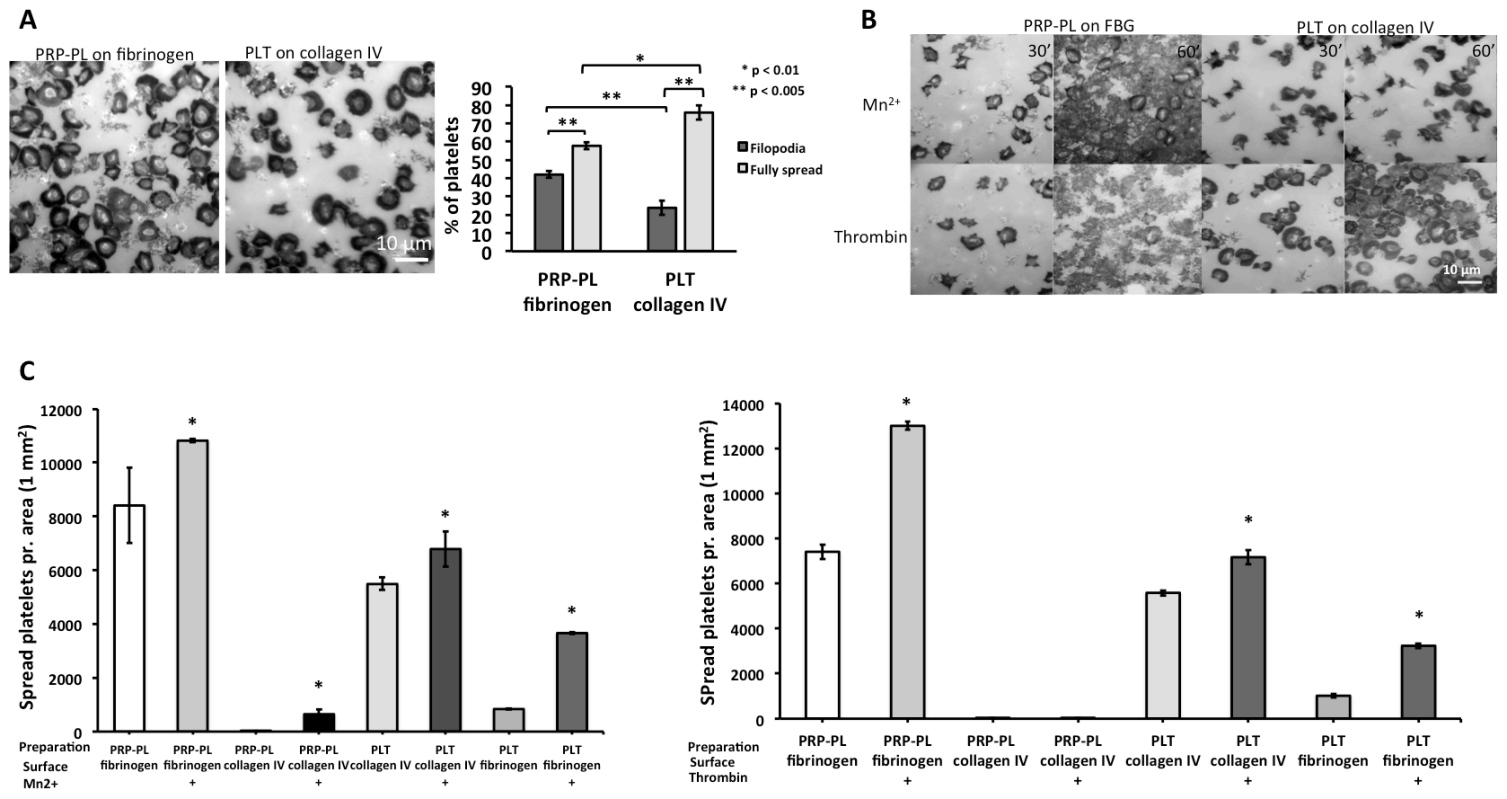
Platelet, PLT and PRP-PL adhesion to collagen IV and fibrinogen with and without Mn ^2+^ and thrombin agonists. ( **A**) PRP-PL on fibrinogen, imaged by IRM (100X magnification). PLT on collagen IV, imaged by IRM (100X magnification). Scale bar: 10 μm. Right graph: % distribution of filopodia and fully spread platelets. Platelet adhesion as a % of filopodia spread platelets vs. fully spread platelets, between PLT on collagen IV and PRP-PL on fibrinogen. Scale bar: 10 μm. Wilcoxon rank-sum test was used, * p-value < 0.005 and ** p-value < 0.01. N experiments=8. N platelets: FBG=532, collagen IV=542. Error bars show Standard Error. ( **B**) Platelets on the surfaces at 30 min. and 60 min. in the presence of manganese (Mn ^2+^) and thrombin. Scale bar: 10 μm. ( **C**) Overview over how many spread platelets per area (1 mm ^2^) of the platelet preparations: PLT and PRP-PL on fibrinogen and collagen IV, in the absence and presence of the two agonist: Left Mn ^2+^ treatment, Right thrombin treatment. Wilcoxon rank-sum test was used, * p-value < 0.05. N experiments=10. Error bars show Standard Error.

**Figure 9.**
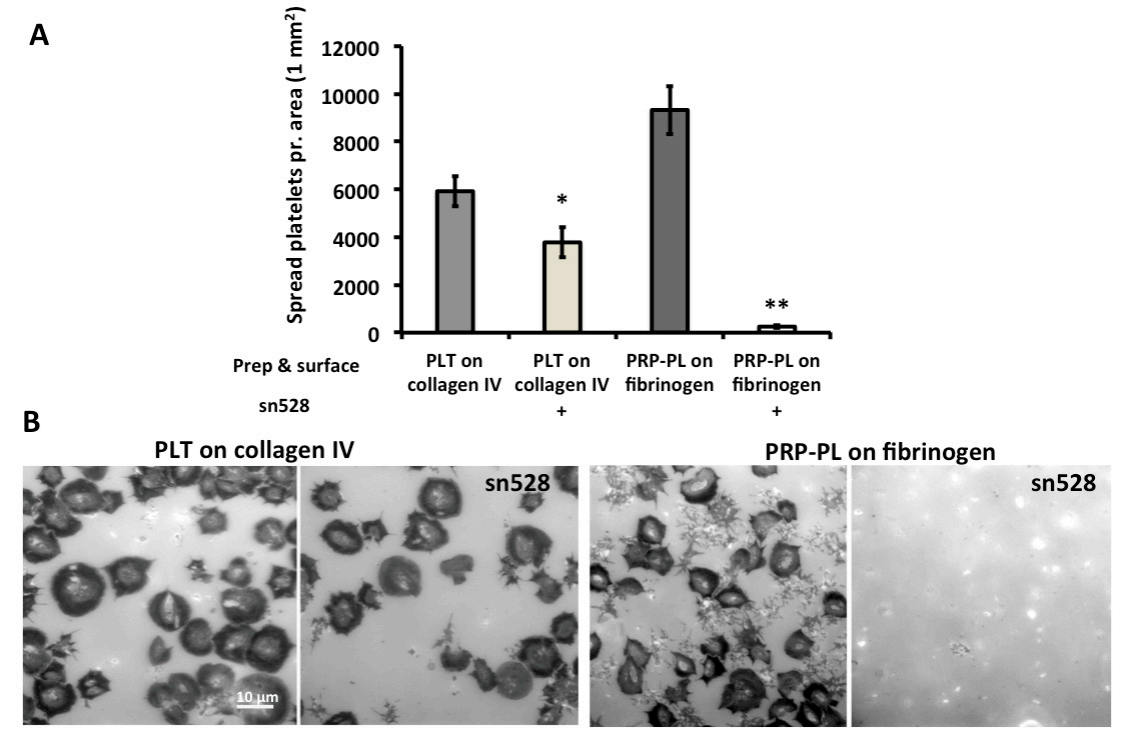
PLT and PRP-PL treated with or without the αIIbβ3 inhibitor sn528, on collagen IV and fibrinogen surfaces. ( **A**) Inhibition of integrin αIIbβ3 by sn528. Effect of integrin αIIbβ3 inhibition shown by platelets on collagen IV and fibrinogen surfaces, with and without addition of the inhibitor sn528 (x-axis). Number of platelets per 1 mm ^2^: y-axis. Wilcoxon rank-sum test was used, * p-value < 0.05 and ** p-value < 0.001. N experiments = 4. Error bars show Standard Error. ( **B**) PLT on collagen IV with and without the addition of the sn528 ligand. PRP-PL on fibrinogen with and without addition of the sn528 ligand. Scale bar: 10 μm.

Beyond the distinct spreading dynamics of PLT and PRP-PL on collagen IV and fibrinogen, their “spreading endpoints” were different. Within 1h after plating, 76% of PLT on collagen completed the filopodia-lamellipodia transition, while only 57% PRP-PL on fibrinogen failed to go through the filopodial-lamellipodial switch ( Figure 8A). To explore the possibility that this arrest is due to insufficient activation of the αIIbβ3-receptor in the PRP-PL, we allowed these platelets to spread on fibrinogen for 30 minutes and then added the platelet activation agonists; Mn ^2+^ or thrombin for additional 30 minutes ( Figure 8B). These experiments clearly indicated that integrin (αIIbβ3) activation drives lamellipodia formation to full spreading ( Figure 8B and C). To test the differential role of integrin αIIbβ3 in the adhesion and spreading on fibrinogen and collagen IV, we seeded PLT and PRP-PL on collagen IV and fibrinogen in the presence or absence of the αIIbβ3 inhibitor sn528 ^32^. This inhibitor had limited effect on the attachment and spreading on collagen while the adhesion to fibrinogen was completely blocked in line with earlier studies ^33^ ( Figure 9A and B).

## Discussion

**Figure 10.**
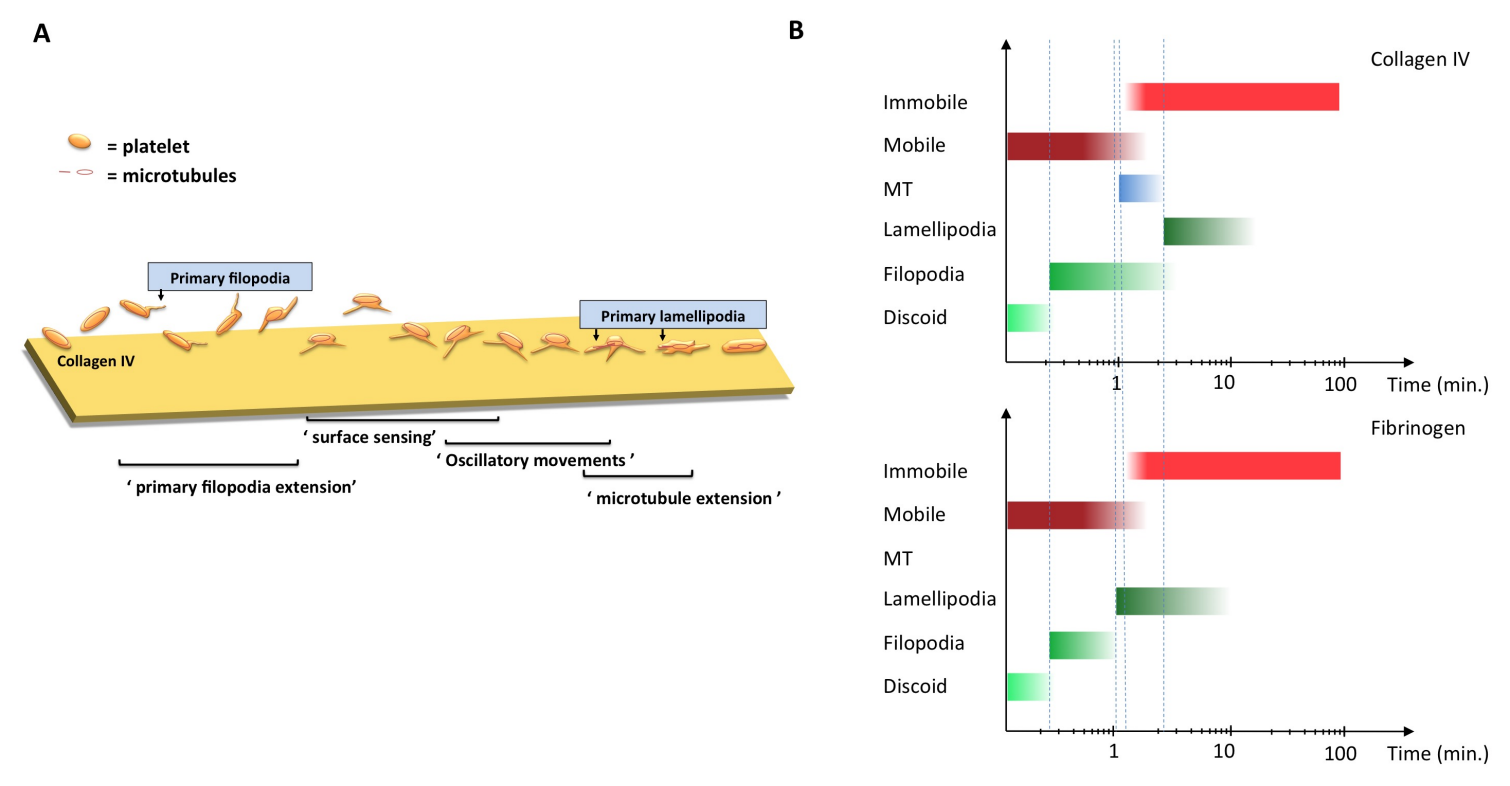
Suggested model for platelet early-stage adhesion. ( **A**) The platelet (in orange) on collagen IV surface is floating as discoid shaped with the microtubule coil intact (red), it then extends filopodia while floating, hereby sensing the surface (yellow; collagen IV) and then exhibits oscillatory movements of attachment and detachment. It then attaches and sends out firmly attached protruding filopodia, one of them containing a dominant microtubule extension in the location where we see the primary lamellipodia forming and then continues to spread. On fibrinogen the attachment process would look similar, with the exception of the protruding MT into a filopodia. ( **B**) Overview of platelet temporal functional interplay for PLT on collagen IV and PRP-PL on fibrinogen. X-axis shows time, y-axis shows a diverse range of parameters under temporal investigation. Red shades correspond to either a mobile or immobile platelet. Blue indicates microtubule polymerization and the shades of green are the different morphological shapes of the platelet phases: discoid, filopodia and lamellipodial. The gradient illustrates if the certain parameter has more or less over all activity.

Our spatiotemporal analysis of platelet spreading demonstrated that platelets sense their environment well before they firmly attach to the functionalized surfaces. During these early spreading stages, the platelets undergo structural changes, manifested by the extension of filopodia, through which intermittent interactions with the surface are established, attenuate the platelets’ mobility, but do not block it ( Figure 10A). These early adhesions, where the platelet changes from a discoid to a spherical shape, and after a short time (<1 min) extends actin-rich filopodia protrusions, followed by lamellipodia spreading was described in the literature, but their dynamic properties, and transition into stable adhesions were not characterized before ^20, 34, 35^. Intermittent adhesions formed during the mobile phase ( Figure 10B), led to immobilization of the adhering platelets, and triggered the subsequent spreading process. The dynamics of platelet spreading during the first minute after reaching the vicinity of the matrix was similar for platelets spreading on collagen or fibrinogen surfaces ( Figure 10B, left from the “1 minute” time point). At this stage the actin ring forms, seemingly by myosin-mediated contraction of actin filaments ^20^. Later events in the platelets’ response appeared matrix-dependent. Filopodial spreading persisted for longer periods and the onset of lamellipodial spreading was delayed on collagen IV. An additional and significant difference is the microtubule extension event, which is apparent only in the cells spreading on collagen. Taken together, these results suggest that distinct environmental signaling cues, induced by collagen IV and fibrinogen, are responsible for the differential late (~ 1–30 minutes) responses of the platelets.

To monitor the early transition of the intermittent adhesions into more stable ones, we have employed and combined real-time DIC and IRM microscopy and developed dedicated image analysis techniques. Our dynamic adhesion data analysis revealed a novel pattern of oscillations, during which the platelet first moves closer to the surface, then further away, then closer again, the so-called tapping events. The frequency of these fluctuations was in the order of ~5–10 seconds, and especially obvious at the filopodial tips. These observations show that early platelet attachment events are oscillatory in nature, and might be coordinated by the platelet itself, yet the mechanism underlying these adhesion oscillations is still unknown. These integrated tapping active events were restricted to the early filopodial spreading stage, leveled out over the course of platelet spreading, and decreased toward the end of the lamellipodial extension period.

Taken together these dynamic adhesion data suggest a model whereby platelets sense the surface through filopodial protrusions while still hovering over the surface (in a time-scale of a few-to-tens of seconds after reaching the surface vicinity). During this the platelet senses the substrate by intermittently touching it or ‘tapping’ on it, before they undergo extensive spreading ( Figure 10A). We hypothesize that this ‘tapping’ process is a rather general mechanism for pre-adhesion sensing, that can play key role in focal complex or focal adhesion formation, as well as in the local organization of cytoskeletal components such as actin and microtubules. In this manner, the local sensing by filopodial protrusions may have a global effect, controlling platelet transformation from an early dynamic phase to a more mature and stable one.

One of the intriguing observations, reported here, is the apparent involvement of microtubules in the regulation of platelets’ spreading on collagen IV. These findings suggest that microtubules play a role in the progression of lamellipodia extension, in line with Waterman-Storer’s observation that microtubules are involved in actin-based protrusions at the leading-edge of migrating fibroblasts ^36^. The absence of lamellipodial protrusions in most platelets adhering to fibrinogen might be attributed to the lack of microtubule-mediated stimulation. Previous studies demonstrated that the interplay between actin and microtubules is bidirectional, and can be directed by specific signaling pathways ^37^. The functional significance of the microtubule extension and its relevance to the regulation of platelet spreading was reinforced by the demonstration that microtubule disruption by nocodazole blocked lamellipodia formation, in spreading PLTs. By the use of high-resolution, close-to-native cryo-electron tomography, we witnessed the microtubules extending only into the base of the filopodia. This stands in contrast with the findings of Patel-Hett *et al.* were they observed EB3-GFP molecules concentrate at the tips of growing filopodial projections ^38^.

When we examined PLT on collagen IV and PRP-PL on fibrinogen, we see that the results indicate striking differences in adhesion dynamics. The primary difference of the two systems is in the receptors mediating the adhesion. In platelets the attachment to collagen IV follow the 2-site 2-step model where the initial attachment and tethering is orchestrated by the GPVI receptor, followed by inside-out activation of integrin α2β1 ^39, 40^. The collagen receptor complex GPVI-FcR γ-chain mediates platelet activation by ligand-mediated clustering of the receptor which triggers an increase in Src family kinases (SFKs) activity and downstream tyrosine phosphorylation of enzymes, adaptors, and cytoskeletal proteins that collectively propagate the signal and coordinate platelet activation in the sense of cytoskeletal remodeling including actin protrusions, degranulation and integrin activation ^41, 42^.

PRP-PL attached to fibrinogen presumably via integrin αIIbβ3, but also via GP-VI, as recent studies have shown ^43, 44^. Integrin αIIbβ3 can be activated by physiological agonists, such as ADP, thrombin or Manganese. When integrin αIIbβ3 is activated it transforms from a low- to high-affinity state. In this manner it binds fibrinogen and other ligands. When a ligand is bound, integrins cluster and the outside-in signaling cascade is stimulated. This drives essential platelet processes, such as attachment, spreading and thrombus formation. The exact mechanism involved in inside-out and outside-in signaling of integrin αIIbβ3 is not fully understood ^45, 46^. What we do know is that when integrin αIIbβ3 mediates outside-in signaling, it induces the tyrosine phosphorylation of multiple proteins. The SFKs plays a major role in these phosphorylation events. SFKs phosphorylate a host of signaling and cytoskeletal-associated proteins in platelets. This includes: phospholipase Cγ2 (PLCγ2), focal adhesion kinase (FAK), and adhesion- and degranulation-promoting adaptor protein (ADAP, also known as SLAP-130), which leads to their recruitment and/or activation ^46^. The partial platelet spreading on collagen IV and especially fibrinogen, were caused by receptor inactivation, which was overcome by treatment with Mn ^2+^ and thrombin. Indicating the necessity of in particular the αIIbβ3 integrin in the full platelet activation response.

It was clear to us that the adhesion to fibrinogen is strictly dependent on αIIbβ3, which we showed could be blocked by the specific inhibitor of this integrin, sn528 ^10^. Addition of this inhibitor to PLT plated on collagen had only limited effect, suggesting that the adhesion in that system is mediated mainly by the generic collagen IV receptors in platelets GP-VI and α2β1. This is consistent with the findings of Thornber *et al.* were they defined the necessity of αIIbβ3 in lamellipodia progression for platelets on collagen related peptide, fibrinogen and thrombin ^33^.

We believe the washed platelets between the surfaces cause differences in adhesion and spreading of PLT, affecting the microtubule filopodial extension, which may play a role in promoting the spreading for platelets on collagen IV. The lack of a MT extension may hinder lamellipodia spreading for PLT on fibrinogen. This would indicate a surface-specific receptor response and downstream signaling either via GP-VI and α2β1 for collagen IV or αIIbβ3 for fibrinogen.

## Conclusions

This study demonstrates the extraordinary capacity of human platelets to differentially sense and respond to distinct physiological microenvironments - collagen IV and fibrinogen. We show that the adhesion and spreading on these surfaces is a highly complex process that combines selective receptor-mediated interactions, and the triggering of downstream cytoskeletal reorganization processes, which reinforce the platelet’s interaction with the external surfaces via specific membrane protrusions – filipodia and lamellipodia. These spatially and temporally coordinated events take place in a time scale of seconds to minutes, and involve integrin αIIbβ3 activation, for platelets adhering to fibrinogen and microtubule-based activation of lamellipodia in platelets adhering to collagen IV.

## Data availability

### Underlying data

Zenodo: IRM raw data (video format) and dataset (csv) supporting platelet attachment to collagen IV or fibrinogen in percentage over time (related to Figure 1), https://doi.org/10.5281/zenodo.3774819 ^47^.

Zenodo: Raw data, temporal profiling for platelet spreading dynamics (related to Figure 3). https://doi.org/10.5281/zenodo.3774823 ^48^.

Zenodo: Raw data for microtubule extension IRM images (videos) and raw data set (csv) (related to Figure 4), https://doi.org/10.5281/zenodo.3774827 ^49^.

Zenodo: Raw data (IRM videos) of Nocodazole experiments (videos) and raw dataset for statistical purposes (csv) (related to Figure 4), https://doi.org/10.5281/zenodo.3774835 ^50^.

Zenodo: Nocodazole experiment low mag images, IRM, raw data. Platelets fixed, imaged by IRM in low magnification for counting purposes. Platelets are either control or treated with nocodazole, https://doi.org/10.5281/zenodo.3774843 ^51^.

Zenodo: Raw data to support percentage of platelets in each morphological state, 1 hour post-platelet seeding (related to Figure 8), https://doi.org/10.5281/zenodo.3774845 ^52^.

Zenodo: Dynamics of platelet spreading over time with/without treatments with manganese and thrombin (related to Figure 8). Raw images of platelets treated with and without Manganese and thrombin (tif, jpegs) and raw data set (csv), https://doi.org/10.5281/zenodo.3774849 ^53^.

Zenodo: Un-cropped and unedited images/movies for all (DIC, movies, cryo-ET, SEM images). https://doi.org/10.5281/zenodo.3773437 ^54^.

### Extended data

Figshare: Differential dynamics of early stages of platelet adhesion and spreading on collagen IV- and fibrinogen-coated surfaces, https://doi.org/10.6084/m9.figshare.c.4944738 ^24^.

This project contains the following extended data:

- **Figure S1. Platelet integrated activity.** Integrated activity of platelets: the mean absolute value |ΔIRM| at every time point. X-axis: Time in seconds. Y-axis: Platelet mean activity. Red dotted lines separate the phases: background, prior to platelet attachment, filopodial spreading phase, lamellipodial spreading phase, and the fully spread phase.
- **Figure S2. Interactions with the surface for collagen IV and fibrinogen.** The number of pixels interacting with the surface over time for the surfaces collagen IV and fibrinogen. Time in seconds.
- **Figure S3. Quantification and image analysis of platelet spreading, based on IRM live imaging for fibrinogen.** (A) Platelet spreading viewed by IRM, and the corresponding focal activity map, ΔIRM _t_ = IRM _t_ – IRM _t+1_. Positive values (yellow) imply local attachment; negative values (blue) imply local detachment (bottom right). One filopodia initially attaching and detaching (black arrow). Scale bar 2 μm (B) Integrated tapping activity of platelets: the mean absolute value |ΔIRM| at every time point. X-axis: Time in seconds. Y-axis: Platelet mean activity. Red dotted lines separate the phases: background, prior to platelet attachment, filopodial spreading phase, lamellipodial spreading phase, and the fully spread phase. (C) Total number of pixels interacting with the surface over time. Time in seconds. (D) Accumulated attachment and detachment over time shown by activity map, yellow means more attachment events, blue means fewer attachment event. Right images, correspond IRM images. Scale bar 2 um.
- **Movie S1**. Shows the accumulated number of transitions from interaction to not interacting with the surface at every pixel over time.
- **Movie S2**. Shows an overlay of the highly active regions on top of the IRM images over time on collagen IV.
- **Movie S3**. Shows an overlay of the highly active regions on top of the IRM images over time on fibrinogen.

Data are available under the terms of the Creative Commons Attribution 4.0 International license (CC-BY 4.0).

## Software availability

IRM spreading dynamics source code available from: https://github.com/assafZaritskyLab/IRM-Spreading-Dynamics

Archived source code as at time of publication: https://doi.org/10.5281/zenodo.3770506 ^21^

License: GNU General Public License v3.0
