# Supplementary material for: Differential dynamics of early stages of platelet adhesion and spreading on collagen IV- and fibrinogen-coated surfaces
Source: F1000Res. 2020 Jul 3;9:ISF-449. Originally published 2020 May 27. [Version 2] doi: 10.12688/f1000research.23598.2 (PMC7281675; doi:10.12688/f1000research.23598.2)
Supplement: Peer review history

# Peer review: Reviewer response for version 2

**DOI:** 10.5256/f1000research.27687.r66354

**Published:** 2020-07-06

**Authors:** Kenneth Yamada

**Affiliations:** 1 National Institutes of Health, National Institute of Dental and Craniofacial Research, Bethesda, MD, USA

Thanks for the helpful clarifications.

Is the work clearly and accurately presented and does it cite the current literature?

Yes

If applicable, is the statistical analysis and its interpretation appropriate?

Yes

Are all the source data underlying the results available to ensure full reproducibility?

Yes

Is the study design appropriate and is the work technically sound?

Yes

Are the conclusions drawn adequately supported by the results?

Yes

Are sufficient details of methods and analysis provided to allow replication by others?

Yes

Reviewer Expertise:

Cell-extracellular matrix interactions, cell migration, branching morphogenesis

I confirm that I have read this submission and believe that I have an appropriate level of expertise to confirm that it is of an acceptable scientific standard.

# Peer review: Reviewer response for version 1

**DOI:** 10.5256/f1000research.26040.r64006

**Published:** 2020-06-10

**Authors:** Ulrich Schwarz

**Affiliations:** 1 Institute for Theoretical Physics, Heidelberg University, Heidelberg, Germany

The authors have performed a quantitative analysis of platelet spreading on either collagen or fibrinogen coated surfaces. The battery of methods applied is pretty impressive: DIC, RICM (also called IRM), FM (with SIR-tubulin), cryoEM, SEM and quantitative image processing. Together these measurements provide interesting new insight into an important part of wound healing. In particular, this study reveals a surprisingly large role of microtubules, which are known to be important for platelet biology due to the marginal band, but here are also shown to insert into actin-based filopodia, which probe the surface in a “tapping” phase and later initiate lamellipodia. This study also shows the important role of different integrins selected by different matrix components (or blocked by plasma components like the VWF), leading to more complete spreading on collagen. All in all this study is an important advance for a quantitative understanding of the platelet spreading part of wound healing.

 There are a few issues that should be addressed in a revision:

1. Platelet activation: the time point cero for the start of spreading seems to be defined by contact with the matrix-decorated surface. Later in the study additional activation by thrombin is mentioned. However, usually platelets are activated by thrombin or ADP at the beginning, and with variable doses. It should be explained why the study has been performed without such design (or if the reviewer misunderstood this part).
2. Relation to marginal band (MB): the new data on the microtubules is intriguing, but the relation to the marginal band is not clear. It is mentioned that the microtubules inserted into the filopodia arise from the MB. Can this be explained in more detail? Is this process regulated, e.g. by changing motor activity in the MB? It is well known that the MB coils after activation (compare e.g. Diagouraga et al. J Cell Biol 204: 177–185, 2014 ^1^) and I wonder if this can be seen in Fig. 4. 
3. Role of actomyosin: coiling of the MB is related to cortical contractility (compare above reference). Moreover it is well known that platelets also form stress fibers (compare Tanaka and Itoh, J Struct Biol 124: 13–41, 1998 ^2^) and recently it has been shown with traction force microscopy that they build up actomyosin-based traction forces with a time delay in regard to area spreading (Hanke et al. Soft Matter 14:6571-6581, 2018 ^3^). For the general understanding of how the different processes are orchestrated during platelet spreading (as summarized in Fig. 10), it would be helpful to also explain the role of actomyosin contractility.
4. Comparison collagen versus fibrinogen: for most of the study these two conditions are treated symmetrically, but important Fig. 2 only shows data for collagen. I suggest to show the same analysis also for fibrinogen, if possible. This way it would also fit better to Fig. 3.

Is the work clearly and accurately presented and does it cite the current literature?

Yes

If applicable, is the statistical analysis and its interpretation appropriate?

Yes

Are all the source data underlying the results available to ensure full reproducibility?

Yes

Is the study design appropriate and is the work technically sound?

Yes

Are the conclusions drawn adequately supported by the results?

Yes

Are sufficient details of methods and analysis provided to allow replication by others?

Yes

Reviewer Expertise:

theoretical biophysics, cellular biophysics, mechanobiology, cell adhesion, cytoskeleton

I confirm that I have read this submission and believe that I have an appropriate level of expertise to confirm that it is of an acceptable scientific standard.

## References

[1] : Motor-driven marginal band coiling promotes cell shape change during platelet activation. *J Cell Biol*.2014;204(2) : 10.1083/jcb.201306085 177-85 10.1083/jcb.201306085 24421335PMC3897189

[2] : Reorganization of stress fiber-like structures in spreading platelets during surface activation. *J Struct Biol*.1998;124(1) : 10.1006/jsbi.1998.4051 13-41 10.1006/jsbi.1998.4051 9931271

[3] : Dynamics of force generation by spreading platelets. *Soft Matter*.2018;14(31) : 10.1039/c8sm00895g 6571-6581 10.1039/c8sm00895g 30052252

# Response

**Published:** 2020-06-25

**Authors:** Benny Geiger

**Affiliations:** Molecular Biology of the Cell, Weizmann Institute of Science, Israel

For Ulrich Schwarz

Thank you Uli for the insightful comments, questions raised and suggestions.

1) Good point, we decided upon this particular set-up because we wanted to observe the platelet behavior before the addition of an agonist (Mn ^++^ or thrombin).

2) Thanks for relating to the marginal band of microtubules. The marginal band may be seen in figure 4 as suggested, and the relevant live-cell imaging is added here. We have found a good example, addressing the question. Please see movie accessible here. We do believe we can see the MB coiling here, with an extending microtubule.

3) We didn't include it in the study. Treatment with Blebbistatin affected the organization of actin and focal adhesion like structures, but the Blebbistatin-treated platelets effect on MT extension was not tested.

4) The data is available as fig. S3, we felt it would be to cluttered to have both in one main figure.

# Peer review: Reviewer response for version 1

**DOI:** 10.5256/f1000research.26040.r64005

**Published:** 2020-06-08

**Authors:** Kenneth Yamada

**Affiliations:** 1 National Institutes of Health, National Institute of Dental and Craniofacial Research, Bethesda, MD, USA

This manuscript provides nicely quantitative descriptions of the early steps of platelet attachment to either collagen IV or fibrinogen. As such, it will provide valuable information for the field and stimulate additional research in this area, e.g., concerning the regulation of filopodial activity during spreading. There are several aspects of this excellent study that would be strengthened by the addition of either limited amounts of new data or at least some additional discussion.

1. This study focuses on platelet attachment to extracellular proteins absorbed to a substrate, presumably modeling the interaction of platelets with basement membrane or a blood clot. Although the interaction of platelets with fibrinogen has been studied extensively in solution after activation by molecules such as thrombin during the process of platelet aggregation, this study seems to focus on interactions with an immobilized substrate. In that regard, the authors might consider that fibrin rather than fibrinogen would be the more physiological substrate. The results of initial attachment and spreading might be different on fibrin, because it is known that platelets can interact via integrin receptors more effectively to fibrin compared to fibrinogen [e.g., see PMID 26867579 and also 29021578 ^1^, ^2^].
  Although this reviewer does not feel it is essential, it would be quite interesting to see whether the key findings concerning platelet attachment to fibrinogen are different when the substrate is fibrin. Alternatively, the authors could discuss this point about the types of substrate in vivo.
2. The differences in response to collagen IV and fibrinogen depending on whether they were in PRP or in a salt solution were extremely dramatic.  One question that should ideally be discussed involves the physiological interpretation of the blocking of adhesion by von Willebrand factor. Does this mean that attachment to exposed collagen IV in damaged basement membranes in vivo is unlikely?  Some additional discussion of these surprising findings would be helpful.
3. The images of apparent surface receptors in Figure 5 are quite impressive. However, it is not clear whether the authors can call them “surface receptors” without some additional information. For example, were morphological differences observed consistent with the folded and open erect configurations of integrins before and after platelet activation?
4. Minor comments:
  A. In Figure 2D, the figure legend refers to “detachment” over time, yet the heat map coding seems to refer to more or fewer attachment events, so how was detachment represented in this figure?
  B. In Figure 4, what were the substrates used for panel sequences A and B?
  C. In Figure 7, and the text, how was a “filopodia spread platelet” defined?
  D. Figure 9A, do the authors happen to have any information on which receptor was used by activated platelets to spread on collagen IV after the treatment with sn528?
  E. In the summary shown in Figure 10, although this is not at all essential, the authors might consider including information on the conditions used for their platelets, specifically PLT vs. PRP-PL; if not practical, this information should be indicated in the figure legend so that readers will not assume that the platelets were compared under identical conditions on the two different substrates.
  F.  The dishes used in this study were presumably from MatTek rather than from MaTek.
  G. Very minor technical questions: Was the concentration of thrombin used actually 0.12 mg/ml?  What was the purpose of the glycerol added to chemically fixed platelets?

Is the work clearly and accurately presented and does it cite the current literature?

Yes

If applicable, is the statistical analysis and its interpretation appropriate?

Yes

Are all the source data underlying the results available to ensure full reproducibility?

Yes

Is the study design appropriate and is the work technically sound?

Yes

Are the conclusions drawn adequately supported by the results?

Yes

Are sufficient details of methods and analysis provided to allow replication by others?

Yes

Reviewer Expertise:

Cell-extracellular matrix interactions, cell migration, branching morphogenesis

I confirm that I have read this submission and believe that I have an appropriate level of expertise to confirm that it is of an acceptable scientific standard.

## References

[1] : The Platelet Integrin αIIbβ3 Differentially Interacts with Fibrin Versus Fibrinogen. *J Biol Chem*.2016;291(15) : 10.1074/jbc.M115.706861 7858-67 10.1074/jbc.M115.706861 26867579PMC4824994

[2] : Strong Binding of Platelet Integrin αIIbβ3 to Fibrin Clots: Potential Target to Destabilize Thrombi. *Scientific Reports*.2017;7(1) : 10.1038/s41598-017-12615-w 10.1038/s41598-017-12615-w 29021578PMC5636895

# Response

**Published:** 2020-06-25

**Authors:** Benny Geiger

**Affiliations:** Molecular Biology of the Cell, Weizmann Institute of Science, Israel

For Kenneth Yamada

Thank you, Ken, for the insightful questions, suggestions and additions to discussion.

 1) We did compare the platelet “2D adhesion” to substrate-attached  fibrinogen- structures where to “3D adhesion” to fibrin fibers. In the former we can see both filopodial and lamellipodial spreading as described in great detail throughout the paper. Platelets spreading on fibrin fibers was predominantly aligned along the underlying fibers, with essentially no sign for lamellipodia. These differences can be seen here *.*

2) We have addressed the point you made in the relevant section of the revised manuscript.

We do not propose that attachment to exposed collagen IV in damaged basement membranes in vivo is unlikely. The difference between the in vivo setting, where flow exists, which reinforces matrix adhesion.  In our work the adhesion occurs under static condition where no or very little attachment occurs. This is attributable to the fact that  VWF-platelet binding is facilitated by the A1 domain of VWF, which binds to the platelet receptor GPIb/IX/V. This binding requires immobilization followed by shear force, in order to be exposed and available for binding. Since we work under static experimental conditions, this binding doesn’t take place.

3) In previous studies, Ohad Medalia’s group conducted gold labeling experiments to localize integrins (Dahan et al. 2018, Tamir et al. 2016) and glycoprotein 1b receptors  (Stivala S, et al. Haematologica 2020) within platelets. They showed that many of the densities observed by cryo-ET, in platelets protrusions, are indeed integrins.  These references were introduced and added to the paper. Moreover, cryo-ET of mouse platelets lacking αIIbb3 (genetically removed) showed no densities around the plasma membrane (Sorrentino et al. in preparations).

All the minor comments were addressed. Specifically:

a) The glycerol was added to the fixation solution in order to provide a protective environment around the sensitive platelet filopodia.

b) The ‘filopodia spread platelet’ is a platelet with filopodia that does not display lamellipodial extensions and stays in this shape for the entire experiment duration.

# Peer review: Reviewer response for version 1

**DOI:** 10.5256/f1000research.26040.r64004

**Published:** 2020-06-01

**Authors:** Sandra Citi

**Affiliations:** 1 Department of Cell Biology, Faculty of Sciences, University of Geneva, Geneva, Switzerland

This is an interesting study on the initial phases of adhesion and spreading of platelets on two different substrates, collagen IV and fibrinogen. The authors report that on collagen IV a short-term phase of filopodial extension is followed by lamellipodia based-spreading, correlating with the insertion of 1-2 microtubules at the core of the filopodium. On fibrinogen, in contrast, there was only partial filopodia-to-lamellipodia transition, and microtubule extension was not observed. The quantification and image analysis of platelet spreading based on interference reflection microscopy, allowing a temporal profiling of attachment vs detachment and cryo-EM data are particularly interesting. The methods are state-of-the art, the results convincing, and the conclusions are sound.

 The results should be discussed in the context of previous work on the distribution of microtubules in resting platelets and platelet filopodia (Patel-Hett *et al.* Blood, 2008 ^1^). 

 Some of the Figures should be improved for clarity. Figure 1: label panels A-C on the sides. Figure 3 D-E: label panels on the side (D- collagen, E-fibrinogen). Figures 7,  8  and 9: please use dot plots and not bar-graphs. Figure 10 label Panels (A and B), and schematic panel (left) for fibrinogen, to make the difference clearer (only collagen is shown).

 In the overview of platelet temporal functional interplay (Fig 10) it is not clear why the lamellipodia shape phase is there, since the filopodia-to-lamellipodia transition is partial?

 Typos: 4°C not 4C° in first paragraph of Methods, by ligand not byligand in 5th paragraph Discussion.

Is the work clearly and accurately presented and does it cite the current literature?

Partly

If applicable, is the statistical analysis and its interpretation appropriate?

I cannot comment. A qualified statistician is required.

Are all the source data underlying the results available to ensure full reproducibility?

Yes

Is the study design appropriate and is the work technically sound?

Yes

Are the conclusions drawn adequately supported by the results?

Yes

Are sufficient details of methods and analysis provided to allow replication by others?

Yes

Reviewer Expertise:

Cell-cell junctions, cytoskeleton.

I confirm that I have read this submission and believe that I have an appropriate level of expertise to confirm that it is of an acceptable scientific standard.

## References

[1] : Visualization of microtubule growth in living platelets reveals a dynamic marginal band with multiple microtubules. *Blood*.2008;111(9) : 10.1182/blood-2007-10-118844 4605-16 10.1182/blood-2007-10-118844 18230754PMC2343595

# Response

**Published:** 2020-06-25

**Authors:** Benny Geiger

**Affiliations:** Molecular Biology of the Cell, Weizmann Institute of Science, Israel

For Sandra Citi

 Thank, Sandra, for the useful comments, suggestions, corrections and thoughts.

 1) We have further addressed, in the discussion MTs organization and dynamics in platelets, with the relevant reference.

 2) We have added labels in fig. 10 (A and B) and we have referred to the difference between collagen and fibrinogen in the figure legend more clearly. Since the figures would be very similar we thought it to be better to address the issue in this manner.

 3) Typos were fixed
